# Bottom-Gated ZnO TFT Pressure Sensor with 1D Nanorods

**DOI:** 10.3390/s22228907

**Published:** 2022-11-17

**Authors:** Ki-Nam Kim, Woon-San Ko, Jun-Ho Byun, Do-Yeon Lee, Jun-Kyo Jeong, Hi-Deok Lee, Ga-Won Lee

**Affiliations:** Department of Electronics Engineering, Chungnam National University, Daejeon 305-764, Republic of Korea

**Keywords:** ZnO, pressure sensor, TFT, nanorod, sensitivity

## Abstract

In this study, a bottom-gated ZnO thin film transistor (TFT) pressure sensor with nanorods (NRs) is suggested. The NRs are formed on a planar channel of the TFT by hydrothermal synthesis for the mediators of pressure amplification. The fabricated devices show enhanced sensitivity by 16~20 times better than that of the thin film structure because NRs have a small pressure transmission area and causes more strain in the underlayered piezoelectric channel material. When making a sensor with a three-terminal structure, the leakage current in stand-by mode and optimal conductance state for pressure sensor is expected to be controlled by the gate voltage. A scanning electron microscope (SEM) was used to identify the nanorods grown by hydrothermal synthesis. X-ray diffraction (XRD) was used to compare ZnO crystallinity according to device structure and process conditions. To investigate the effect of NRs, channel mobility is also extracted experimentally and the lateral flow of current density is analyzed with simulation (COMSOL) showing that when the piezopotential due to polarization is formed vertically in the channel, the effective mobility is degraded.

## 1. Introduction

Semiconductor based micro-electro-mechanical-systems (MEMS) pressure sensors are attracting a lot of attention in the medical field due to their advantages such as miniaturization, light weight, and low power consumption [1]. High sensitivity pressure sensors are essential for the development of promising bio-applications such as human-machine interaction, wearable and flexible electronic skin (E-skin) using tactile sensors, and various human imitation technologies [2,3,4,5]. It even invades blood vessels to monitor pressure internally through the benefits of miniaturization [1].

Important parameters for sensors are sensitivity, linearity, response time and recovery time, detection limit, selectivity, stability, and reproducibility [6]. As MEMS pressure sensors become smaller, the sensing area decreases. Moreover, most of the pressures detected in the human body are in the low range. A normal adult mean blood pressure range is 120/80 mmHg (16/11 kPa), intraocular pressure range is 10~21 mmHg (1~3 kPa) and intracranial pressure range is 5~15 mmHg (0~2 kPa) [7,8,9,10]. For the sensor to use these small pressures as inputs, the sensitivity becomes more important. The high sensitivity MEMS pressure sensors can solve this problem combining a new process of amplifying a signal for better recognition.

The main goal of this study is devising a high-sensitive pressure sensor with a new structure that can amplify small pressure signals while using human-friendly materials suitable for bio-applications based on piezotronic. Generally, the piezoelectric materials are capable of interconversion of mechanical and electrical energy. When their structure is deformed by an external force, the positive and negative charges are separated and the dipole is formed, creating a piezopotential [11,12]. On the contrary, the piezotronic material have a coupled effect that interacts the piezoelectric and semiconducting properties and resistance change is induced by this polarization [13,14,15]. The piezoelectric materials include ZnO, PZT, BiFeO_3_, LiNbO_3_ and AlN. ZnO is the most suitable for the purpose of this study because it has advantages of biosafety, non-toxicity, high piezoelectric constant and coefficient with simultaneous semiconducting properties [16].

As shown in Table 1, previously reported pressure sensors have the following structure: vertical film type [17,18], vertical nanostructure type [19,20,21] with top and bottom electrode; lateral film type [22,23,24], lateral nanostructure type [25] with anode and cathode electrode. Since it was first reported by Wang, X. D.’s group in 2006 [11], materials with semiconducting properties among piezoelectric materials have been used as electronic transports, and the study has extended to a lateral structure from the previous vertical structure with piezoelectric ceramic.

In many cases, a vertical structure is favored to use the vertically generated piezopotential without any losses. However, the lateral structure has an inherent advantage that it can be extended to the TFT structure. Although various structures have been reported, when examining state-of-art research trends, there have been few studies using nanostructures as a medium for pressure amplification or fabricating piezotronic pressure sensors with TFT structures as in our work [26,27,28,29,30]. This study proposes a ZnO NRs-based TFT pressure sensor with a lateral structure for sensitivity enhanced pressure sensors. This device has the attractive advantage of power consumption saving as well as sensitivity improvement. The experimental results of this research show that the sensitivity is improved through the NRs growth and the gate voltage control of TFT. The power consumption of ZnO sensor, which operates in the depletion mode (normally on), is decreased through the on/off switching operation of TFT. In conclusion, this paper can show performance improvement by improving sensitivity and reducing power consumption through NR growth and TFT structure. Also, the operation mechanism of this device is interpreted as a piezotronic effect, and another possible mechanism is proposed in Section 3.3.

The architecture of the paper is organized as follows: Section 2 describes the fabrication and characteristic evaluation method of the experimental device. Section 3 discusses the results and analysis. Physical analysis using XRD and SEM, and electrical analysis to investigate the nanorods effect and TFT gate effect were analyzed. At the end, ‘mobility degradation’ as a possibility to interpret this was discussed through COMSOL simulation and TFT parameter extraction. Finally, Section 4 presents our conclusions.

## 2. Experiment

### 2.1. Device Fabrication

**Figure 1:**
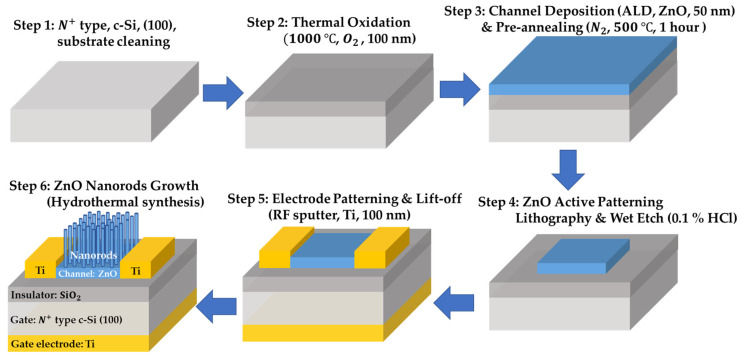
ZnO NRs-based TFT pressure sensor; schematic illustration of the suggested device structure and process flow. The thin film type does not undergo pre-annealing and ZnO NRs growth. The film annealing type omits only the hydrothermal synthesis.

The proposed ZnO NRs-based TFT pressure sensor was fabricated. Figure 1 shows the device structure schematic and process flow. A prime graded $n^{+}$ c-Si (100) substrate was used as a back gate. To grow gate oxide 100 nm, thermal oxidation was performed at 1000 °C in an $O_{2}$ atmosphere for 1 h. The channel ZnO (40 nm) was deposited using atomic layer deposition (ALD). To compare the sensitivity according to the absence or presence of nanorods, thin film type and NRs stacked type were investigated. Thin film devices leave out pre-annealing and NRs growth process. For NRs stacked device fabrication, NRs were formed by hydrothermal synthesis on the ZnO film as a seed layer after 500 °C annealing in $N_{2}$ ambient for 1 h. In order to verify the nanorod sensitivity improvement effect, the ZnO thin film annealing type was also analyzed. To define the active region, the ZnO layer was wet etched with HCl:DI-water solution. After forming the Ti (100 nm) source and drain using RF sputter, lift-off was conducted, and a Ti back gate electrode was also formed for better contact on the back side of the Si substrate.

### 2.2. Hydrothermal Synthesis

Among the methods for forming nanorods, hydrothermal synthesis is low-cost and can be mass-produced [31,32], and the morphology (height and diameter) of NRs can be manipulated by controlling parameters (molarity, temperature and time) [33]. In this study, a ZnO thin film was used as the device channel and seed layer, and NRs were grown on it by the hydrothermal growth method. Before growing NRs, the as-depo ZnO thin film was annealed at 500 °C in $N_{2}$ atmosphere for 1 h. The condition of synthetic solution was that 0.05 M of hexamethylenetetramine (HMTA, ≥99.0%) and 0.05 M of zinc nitrate hexahydrate $(\mathrm{Zn}{\left({\mathrm{NO}}_{3}\right)}_{2}\cdot 6H_{2}O$, ≥99.0%) were used at the same ratio. This reaction was conducted under 363 K with foil sealing on the beaker using a hot plate for 40 min, and a steering bar was used during the growth reaction for a steady flow of the synthesis solution. As an advanced step, the density of NRs was controlled by changing the synthesis parameters and using the mesh pattern shadow mask [34,35]. This shows that the denser the nanorods (occupies a large area or the greater the number), the greater the effect of improving the sensitivity. Table 2 includes a detailed synthesis condition for controlling the density of ZnO NRs.

### 2.3. Characterization

To analyze the effect of NRs in the piezoelectric based pressure sensor, various weights of the same area were forced on the device. The sensing area was $\;0.5\times 0.5\;{\mathrm{cm}}^{2}$ and a pressure (or weight) was applied by placing very small weight screws on a tray having an area of $1\times 1\;{\mathrm{cm}}^{2}$. To check the gate effect of the suggested ZnO NRs-based TFT, a bottom gated TFT having a sensing unit of $100\times 100\;{\mu m}^{2}$ was fabricated. In this test, the pressure was turned on and off using a floating tungsten micro size tip. All electrical analyses were performed using a 4155B semiconductor parameter analyzer. In our experiment, simulation (COMSOL) is also performed to illustrate the internal field effect in the device where the piezopotential is simulated as a gate voltage during the loading state. When the piezopotential occurs vertically in the channel, changes in lateral current density is analyzed.

## 3. Results and Discussion

### 3.1. Physical Characteristic Analysis

**Figure 2:**
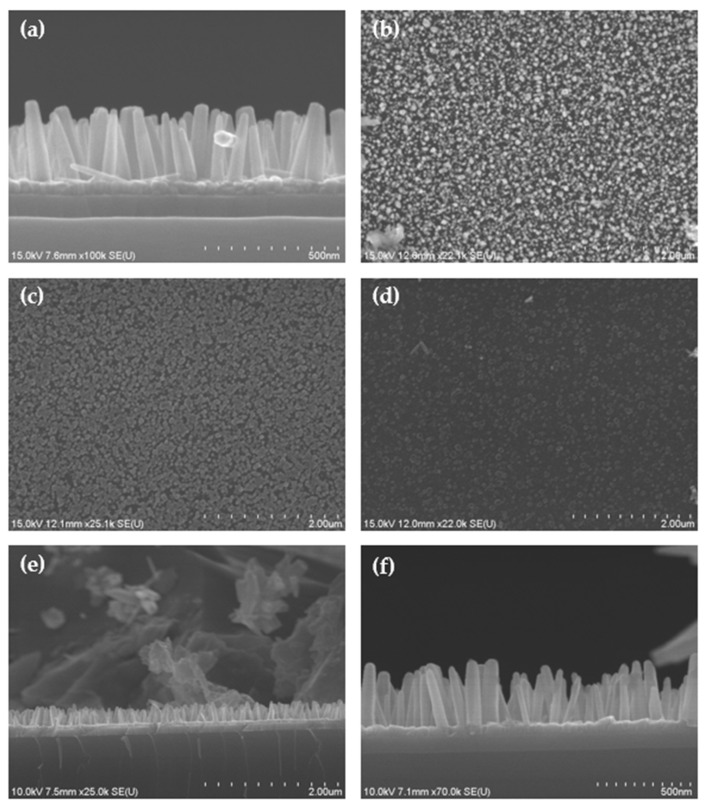
Nanorod SEM image of the fabricated device: (**a**) Cross section of NRs stacked: High density (0.05 M). Average diameter 60 nm and height 400 nm; (**b**) Top view of NRs stacked: High density (0.05 M); (**c**) Top view of NRs stacked: High density (0.050 M). By reducing the molarity, a change in density with decreasing diameter was expected, but there was no significant change in density with 0.05 M; (**d**) Top view of NRs stacked: Low density (0.05 M). During hydrothermal synthesis, the mesh pattern shadow mask was covered. By intentionally reducing the amount of source in contact with the seed layer, the density was reduced by interfering with nanorod growth; (**e**) NRs SEM image after 5~120 g loading test over 200 cycles. It showed that it was not broken; (**f**) SEM image of unbroken NRs after 450 g heavy weight durability test.

A scanning electron microscope (SEM) was used to identify the nanorods grown by hydrothermal synthesis. As shown in Figure 2a, the 1D nanorods are formed on a planar channel of ZnO to improve the sensing ability. This shows NRs hydrothermally synthesized at 0.05 M (NRs stacked: High density (0.05 M)) on a Si substrate on which 100 nm ${\mathrm{SiO}}_{2}$ is grown, 40 nm of ZnO is deposited thereon. The NRs grown under these conditions have a diameter of about 60 nm and a height of about 400 nm. It can be confirmed from the image that the nanorods are predominantly grown in the C-axis direction. We are also fortunate to be able to observe the contact surface of a single isolated nanorod, which has a hexagonal shape. Through this, it was confirmed that the nanorods were successfully grown. Figure 2b–d are SEM images of the result of controlling the density of NRs by manipulating the hydrothermal synthesis conditions. In all three conditions, the ratio, temperature, and synthesis time were the same; Figure 2b,c predicted the density change according to the decrease in the diameter of the nanorods as the molar concentration decreased. However, our SEM results did not show much significance in the density change. Therefore, the sensitivity improvement in Figure 2b,c was also similar. On the contrary, in Figure 2d, all the synthesis conditions are the same as in Figure 2b. Instead, the density was reduced by intentionally blocking the flow rate of the synthetic solution serving as the source in contact with the ZnO surface. The detailed discussion of the sensitivity improvement depending on density is described in Section 3.2.

**Figure 3:**
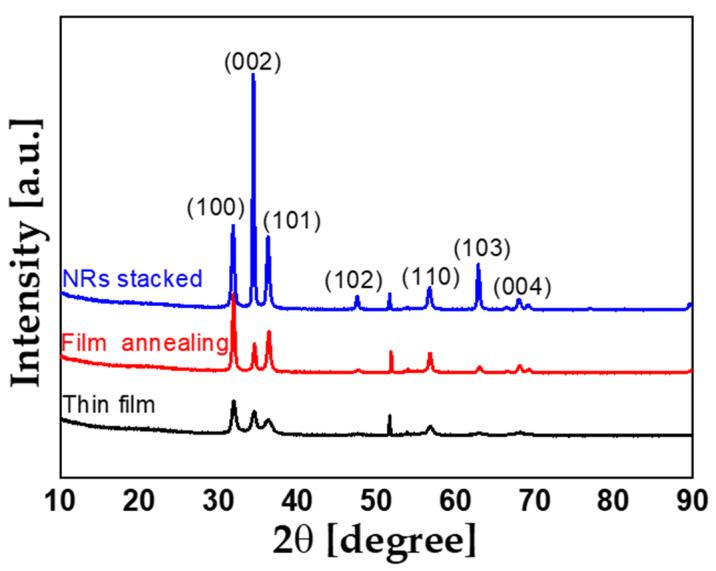
XRD data of ZnO (40 nm) thin film, film pre-annealing, NRs growth type devices; The (002) peak was remarkably increased in the NRs stacked type device grown by supplying a source of ZnO nanorods through hydrothermal synthesis.

X-ray diffraction (XRD) was used to compare ZnO crystallinity of thin films, film annealing, and NRs stacked devices. As shown in Figure 3, the crystallinity of the ZnO thin film shows a slight change through the pre-annealing process. Having a high C-axis orientation along the (002) plane is highly correlated with piezoelectric activity [36]. NRs have a strong tendency to grow along the C-axis [37]. As a result of XRD analysis, the (002) peak of ZnO was remarkably improved when NRs were deposited on the thin film than when the film was simply annealed. This means that the piezoelectric properties are strengthened, and more dipoles are generated when the same pressure is applied to the ZnO channel.

### 3.2. Electrical Characteristic Analysis

#### 3.2.1. ZnO Nanorods effect

**Figure 4:**
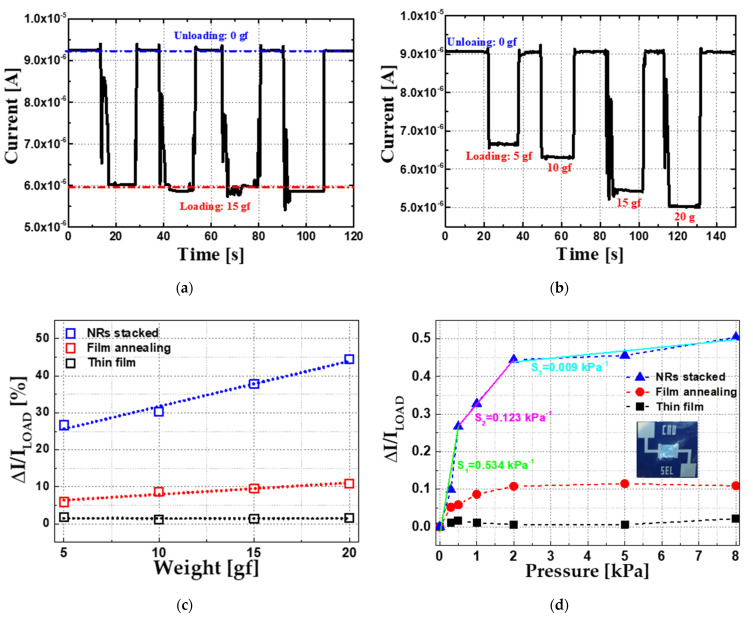
Pressure test results of fabricated devices; (**a**) NRs stability test. 15 g loading results after 200 cycles of NRs stacked type; (**b**) Time dependent current result of NRs stacked type. The amount of change in current was measured by repeatedly raising and lowering an object of 5~20 g; (**c**) Weight dependent current ratio result in a small range (5~20 g); (**d**) Sensitivity measurement up to 8 kPa. ($S_{1}:\;0\sim 0.5$ kPa, $S_{2}:\;0.5\sim 2$ kPa $S_{3}:\;2\sim 8$ kPa). Inset is pressure sensor device with a wide sensing area for various weight measurements.

Figure 4 is the measurements of various weight loading tests of NRs type. Figure 4a shows the operational stability of the NRs device after the cycle test. In Figure 4b, the weights of 5~20 g were sequentially loaded. As the weight was applied, the current decreased linearly. It was observed that the fabricated ZnO pressure sensors increased their resistance when pressure was loaded. This deteriorated conductance can be explained by mobility degradation based on the piezotronic effect in the lateral ZnO pressure sensor, which will be considered in Section 3.3. Figure 4c is the measured unloading current $(I_{UNLOAD}$) and loading ($I_{LOAD}$) ratio, and sensitivity was calculated based on Equation (1), where ∆I is subtraction of loading and unloading current and ∆P is change in pressure.

(1) $$Sensitivity\;\left(S\right)=\frac{∆I/I_{LOAD}}{∆P}\;\left[{\mathrm{kPa}}^{-1}\right]$$

To compare the sensitivity according to the presence or absence of nanorods, the thin film type and the NRs stacked type were compared, and the film annealing type device was also investigated to distinguish it from the effect of pre-annealing in the process of fabricating the NRs device. According to the data in Figure 4c, the response current ratio result of stacking NRs on the ZnO channel is 26~44%. This is much better than 1~2% of the thin film type, and is better than 25% reported by Lee, C. T.’s group in 2015 [38]. In the case of the annealed film type the sensitivity is improved by 6~20% and this is because as the crystallinity of the ZnO channel is improved through the pre-annealing process, the piezoelectric properties get better leading to enhance the sensitivity. So, large ratio of response current is very important to increase sensitivity. In the NRs stacked type, the sensitivity is remarkably improved and can be predicted through the simple physical formula of *P* = *F*/*A* calculating a pressure (*P*) when a force (*F*) is on the force transmission area (A).

In the suggested device structure, NRs transmit a vertically applied force to the sensing unit. NRs have a smaller area for transmitting pressure compared to the thin film type sensor. Therefore, when the sensor is pressed with the same force, the NRs stacked device receives the amplified pressure.

Table 3 shows the sensitivity and response current ratio according to the density of NRs. Compared to the thin film type, the annealing type is slightly improved. The NRs stacked type showed better sensitivity than the others. The high density had better output response current ratio than the low density. NR stacked: (0.05 M) and (0.005 M) have similar densities, and the measurement results are also similar. The larger the area occupied by the nanorod, the higher the output.

**Figure 5:**
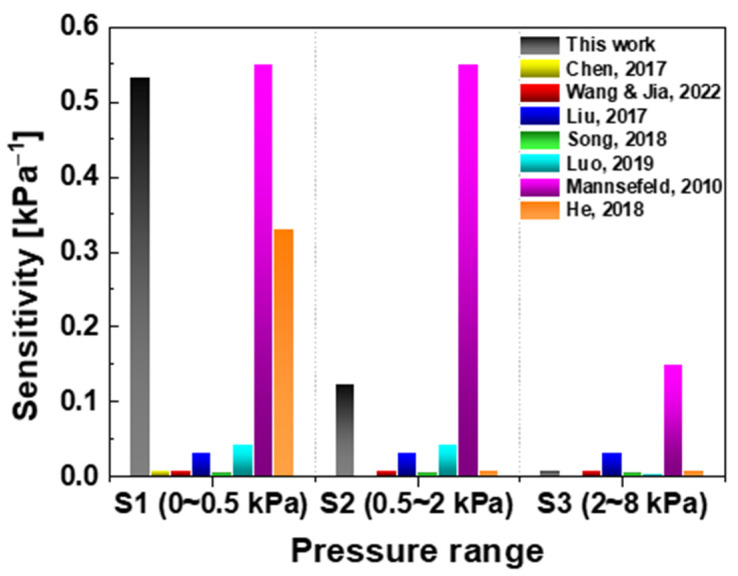
Comparison of sensitivity between previously reported MEMS pressure sensors and this study; As compared to the reported semiconductor-based pressure sensors, this work device does not lag behind the previous research showing excellent performance. The pressure ranges are $S_{1}:\;0\sim$0.5 kPa, $S_{2}:\;0.5\sim 2$ kPa $S_{3}:\;2\sim 8$ kPa. [39] Chen et al., (2017). [40] Wang et al. [41] Jia et al., (2022). [42] Liu et al., (2017). [43] Song et al., (2018). [44] Luo et al., (2019). [45] Mannesfeld et al., (2010). [46] He et al., (2018).

In Figure 5, when the sensitivity of this study and the reported pressure sensors were compared, the performance was not inferior. If the output linearity is improved in future work, it is expected that best sensitivity will be obtained.

#### 3.2.2. TFT Gate Effect

**Figure 6:**
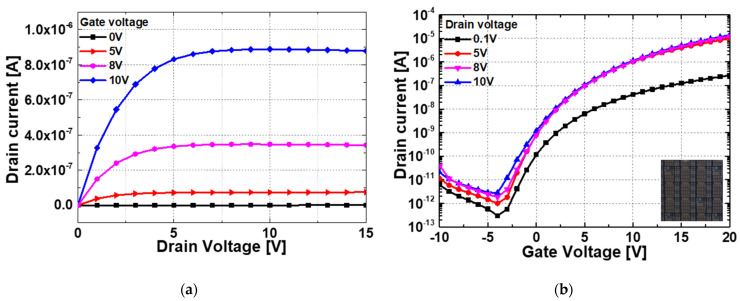
The I-V characteristics of fabricated TFT pressure sensor; (**a**) A plot of $I_{DS}-V_{D}$, (**b**) $I_{DS}-V_{G}$.

**Figure 7:**
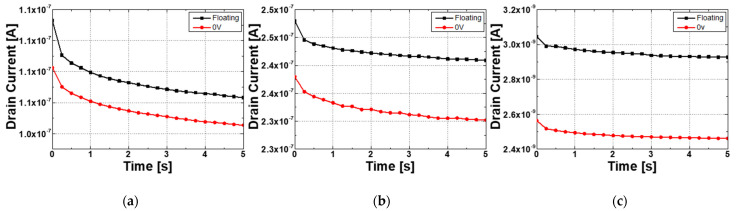
Power consumption test result of the fabricated ZnO TFT device. The black line is the floating gate voltage, and the red line is 0 V biased to the gate; (**a**) Thin film type; (**b**) Film annealing type; (**c**) NRs stacked type.

To investigate the effect of the TFT gate in a pressure sensor with a lateral structure, a device with a width (W) of 100 μm and a length (L) of 100 μm was fabricated. Figure 6 shows the I-V characteristics of this TFT. As can be seen from the results of the drain current ($I_{DS}$) according to the gate voltage ($V_{G}$), the ZnO device is turned on in the negative voltage region. In other words, the ZnO device operates in depletion mode, which is always on at 0 V. By varying the gate voltage, the resistivity of ZnO can be controlled without any special doping techniques. Figure 7 investigates I_DS_ with the gate floated like a 2-terminal device and when 0 V is applied in a 3-terminal device. It is shown that the stand-by current can be reduced by applying the gate voltage especially in depletion mode TFT, which means that the stand-by power consumption will be saved by applying the gate voltage. Also, the gate sweep voltage is independent of the power composition as the gate input impedance is infinite.

**Figure 8:**
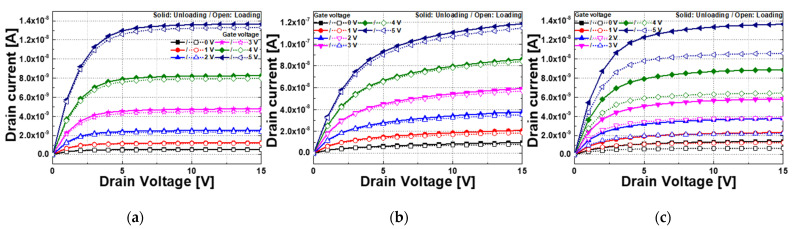
Pressure test result on the manufactured TFT pressure sensor. A solid is unloading condition and an open is the loading condition of the corresponding gate voltage; (**a**) Thin film type; (**b**) Film annealing type; (**c**) NRs stacked type.

**Figure 9:**
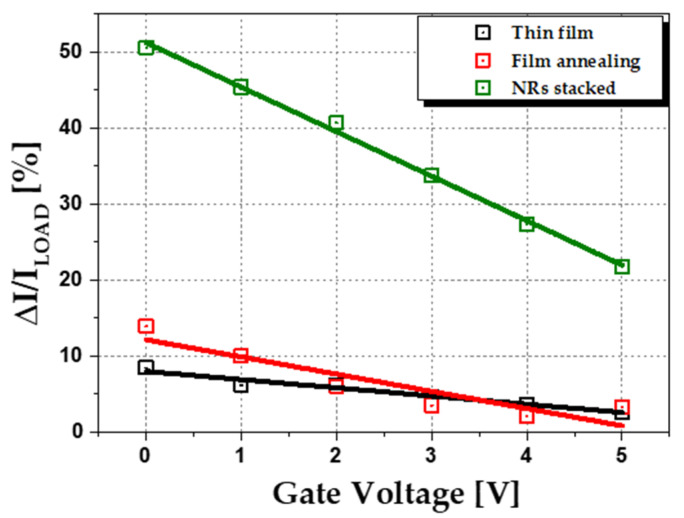
Ratio of response current results according to gate voltage extracted from the measurement of the fabricated ZnO TFT pressure sensor.

Figure 8 is the I_DS_ vs. V_DS_ of the loaded and unloaded device and I_DS_ changes according to the pressure. The solid symbol is the loaded state, and the open symbol is the unloaded state. In all cases, the current decreased when pressure was applied. The amount of current reduction at the NRs stacked type is larger than that of the thin film type. When the same pressure is applied, a large change of response current means high sensitivity. Because the ZnO was more deformed, more piezoelectric effects occurred, which induced greater resistance. Figure 9 and Table 4 are the result of output enhancement tests due to the proposed structure. NRs stacked type had a significantly larger response current ratio than the others, and in all types, as the gate voltage decreased, the response current ratio increased. The cause of this will be discussed in Section 3.3.

Also, due to the NRs effect, about 6~7 times higher response current ratio was obtained in the NRs stacked type compared to the thin film. The film annealing type is slightly improved over the thin film type. This shows a similar tendency to Section 3.2.1.

### 3.3. Sensing Mechanism: Mobility Degradation

**Figure 10:**
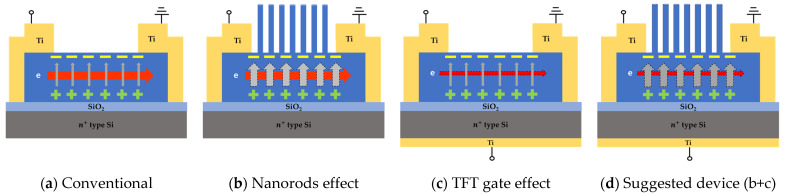
Schematic illustration of mobility deterioration sensing mechanism of lateral ZnO piezotronic pressure sensors. The dot outline indicates the changed component due to the effect; (**a**) Conventional thin film ZnO pressure sensor; (**b**) Nanorods effect. The vertical E-field is enlarged due to the amplified pressure; (**c**) TFT gate effect. The lateral current flow in the channel is intentionally weakened by the gate voltage; (**d**) Suggested device. Both NRs effect and TFT gate effect can be used to achieve improved sensitivity and reduced power consumption.

The measurement results can explain that lateral ZnO piezotronic pressure sensor has conductivity degradation under loaded state. In piezoelectric phenomenon, the reason is explained by that ZnO has an asymmetric wurtzite structure. When the structure is deformed due to external stress, there is a difference between the movement of + charged ${\mathrm{Zn}}^{2+}$ and—charged $O^{2-}$, so the neutral breaks and surface charges occur [47,48]. This polarization causes E-filed in the vertical direction in the ZnO lateral channel, and this force interrupts the flow of electrons moving in the lateral (source to drain) direction and reduces mobility. Figure 10a shows that when pressure is applied to the conventional ZnO pressure sensor, the resistance of the channel increases and the current decreases.

Table 5 is the results of extracting the parameters of the TFT pressure sensors used in this study (W: 100, L: 100 μm). The threshold voltage $\left(V_{th}\right)$ was extracted using the CC (constant current) method. [49]. Subthreshold swing (SS) and TFT turn on/off switching current ratio ($I_{on/off}$) were also calculated. Field-effect mobility $\left(\mu_{FE}\right)$ was extracted by the method of Park. J. S’s group published in 2007 [50]. According to the results of Table 5, the field-effect mobility decreases when pressure is applied. This is consistent with the result of increasing resistance when loading weight, as shown in Figure 4 and Figure 8. To further prove this, a COMSOL simulation was performed.

**Figure 11:**
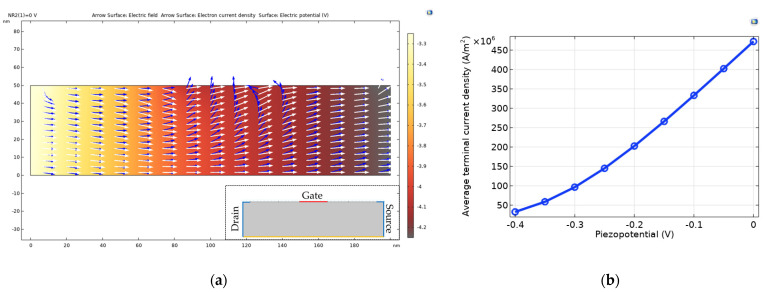
COMSOL simulation for validation of mobility change; (**a**) Lateral current density change by vertical E-field in the ZnO. Drain-source was defined at both ends like an inset. To simulate the polarization at the ZnO channel interface, the gate electrode was defined on the top and it means the place where the nanorod was locally grown; (**b**) Results of current density flowing through the ZnO channel when a gate voltage (piezopotential) is applied. As the polarization increased, the current decreased.

Figure 11 is the result of COMSOL simulation. As shown, Figure 11a is a simulation of the pressed ZnO channel. The drain and source were defined like an inset and the gate electrode was defined locally on the top, which means locally grown nanorods. Through this, it was simulated that polarization occurred at the interface of the ZnO channel when pressure was loaded. In this simulation, it is assumed that a negative charge is formed on the top of the ZnO channel by applying a negative voltage to the gate electrode. While the drain voltage of 1 V was fixed, the current change according to the change of the gate voltage was measured. In Figure 11a, the blue arrow is the vertical electric field induced by the polarization. The white arrow is the electron current density flowing lateral in the channel. When the piezopotential due to polarization was formed vertically in the channel, the lateral flow of current density was disturbed. This means that the channel mobility decreases when pressure is applied.

Polarization occurs at the position where the nanorods are formed and pressed, and the current flow is distorted at the position where the vertical E-field is induced. Through this, the deformation of the ZnO structure changes depending on the pressure, the amount of dipole generation changes, and the piezopotential changes, which leads to deterioration of mobility and change in resistance. As a detailed condition, before defining the gate electrode, it was isolated from the drain-source by using a thin insulator gate under it. The oxide relative permittivity of the thin insulator was 4.5 and the thickness was 120 nm. Figure 11b explains this well. As shown, −0.4 V to 0 V was applied to the gate electrode (step is 0.05 V). The voltage applied to the gate voltage means the piezopotential generated in the ZnO channel. It can also be seen from this simulation result that the flow of lateral electrons decreases as the voltage generated by the polarization increases.

Therefore, the sensing mechanism of the proposed piezotronic pressure sensor is as follows: When pressure is applied, a vertical E-field (piezopotential) is generated by polarization, which impedes the lateral current flow in the channel and increases its resistance. In other words, sensing is performed according to how much it is affected by the vertical E-field reactively. When the same input pressure is given, the sensitivity can be improved depending on how sensitively the lateral current flow responds to the vertical E-field. As a way to increase the influence of the vertical E-field, we propose two methods. Growing nanorods on the channel and using bottom-gate TFT.

#### 3.3.1. Nanorods Effect on Mobility Changes: Vertical E-Filed Enhancement

Figure 10b shows the NRs effect. NRs transmit pressure locally to the sensing unit, and the deformation occurs more by the amplified pressure. As a result, the vertical E-field becomes larger than that of a thin film without NRs. As shown in Figure 2a, the shape of the NRs has a smaller force transmission area compared to the film type, so greater pressure is transmitted, even if the same force is applied. That is, the transfer pressure can be amplified by reducing the area even at the same force, according to the relation of *P* = *F*/*A* where *P* is the pressure, *F* is the external force and A is sensing area. In addition, ZnO nanorods grow in the C-axis and crystallinity in the direction (002) is dominant. This improves the crystallinity of the channel and the piezoelectric material, ZnO. As a result, the offset of polarization is reduced, and piezoelectric properties are also improved. In conclusion, by growing the NRs on the planar channel, the vertical effect is enhanced, which induces a larger change in the lateral current even under the same pressure load.

#### 3.3.2. TFT Gate Effect on Mobility Changes: Lateral Current Flow Degradation

Figure 10c shows the gate effect of TFT. While traditional sensors are two-terminal devices, the suggested device has three terminals. It has a new merit that the conductivity control of the channel is possible. By adjusting the gate voltage, the flow of lateral current is intentionally weakened. A weakened lateral current flow seems to respond more strongly to the vertical E-field. Through this, even when the same piezopotential of the same pressure is generated, it can be detected more sensitively.

In addition, this has the additional advantage of lowering power consumption by reducing the leakage current in the stand-by mode. It is difficult to apply a doping technique to control the resistivity of ZnO. However, the TFT-structured ZnO pressure sensor can normally be turned off by adjusting the resistance in the channel due to the role of the gate.

## 4. Conclusions

In this study, a ZnO NRs-based TFT pressure sensor is proposed, and ‘mobility degradation’ is suggested as another possible mechanism of the piezotronic effect sensor. It responds sensitively to stimulation through pressure enhancement with NRs by a small area effect. The NRs amplifies the transmitted pressure when a force is applied. The sensitivity is improved by about 16~20 times compared to the thin film type device. In the suggested device, the lateral current flow is deteriorated under the loaded state by the vertical E-field which is caused by piezoelectric phenomenon. The device has a three-terminal TFT structure and it becomes possible to control the electrical conductivity of the sensor. By adjusting the gate voltage, the lateral current is intentionally weakened and the sensitivity to the input is improved. Moreover, the stand-by current of the ZnO device operating in depletion mode can be reduced by the gate voltage, which is in favor of the power consumption. Mobility degradation was proposed to explain all these results. The polarization creates a weak vertical E-field in the channel and blocks the flow of electrons moving to the lateral, increasing the resistance. This was reviewed through COMSOL and field effect mobility extraction. This work can contribute to the development of semiconductor-based MEMS pressure sensors that are harmless to the human body, improve sensitivity at low pressure range with 1D nanorods and reduce stand-by power consumption with TFT structure.

## Figures and Tables

**Table 1:** Previously reported structure of MEMS presser sensor based on piezoelectric mechanism.

	Vertical	Lateral
Thin film type	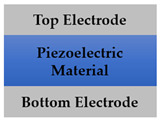	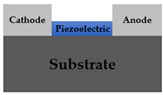
	[17] Polymide/ZnO Piezoelectric (2003) [18] PVDF-TrFE Piezoelectric (2012)	[22] ZnO Piezotronic (2013) [23] AlGaN/AlN Piezotronic (2015) [24] GaN/Graphene Piezotronic (2020)
Nanorods stacked type	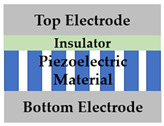	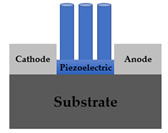
	[19] Al_2_O_3_/ZnO NWs Piezoelectric (2014) [20] ZnO NWs/MgO Piezotronic (2015) [21] Seedless ZnO NRs piezoelectric (2020)	[25] ZnO NWs/Graphene Piezotronic (2020)

**Table 2:** Hydrothermal synthesis condition.

Device Types		Synthesis Conditions			
	Pre-Annealing	$Zn{\left(NO_{3}\right)}_{2}\cdot 6H_{2}O$	HMTA	Temperature	Time
Thin film	-	-	-	-	-
Film annealing	$N_{2}$, 500 °C, 1 h	-	-	-	-
NRs stacked: (0.05 M)	$N_{2}$, 500 °C, 1 h	0.05 M	0.05 M	90 °C	40 min
NRs stacked: (0.005 M)	$N_{2}$, 500 °C, 1 h	0.005 M	0.005 M	90 °C	40 min
NRs stacked with shadow mask: (0.05 M)	$N_{2}$, 500 °C, 1 h	0.05 M	0.05 M	90 °C	40 min

**Table 3:** Comparison of sensitivity and response current ratio with previous studies.

Device Types	Material	$∆I/I_{0}\;\left[\%\right]$	$\mathrm{Sensitivity}\;\left[kPa^{-1}\right]$
Thin film	ZnO	8	$S_{1}:\;0.034$, $S_{2}:0.006$ $S_{3}:0.002$
Film annealing		10	$S_{1}:0.116$, $S_{2}:0.003$ $S_{3}:0.001$
NRs stacked: (0.05 M)		53	$S_{1}:0.534$, $S_{2}:0.123$ $S_{3}:0.009$
NRs stacked: (0.005 M)		49	-
NRs stacked with shadow mask: (0.05 M)		25	-
Piezoelectric [39]	PDMS/Graphene		$S_{1}:$0.009
Piezoresistive [40,41]	Graphene	-	$S_{1}\sim S_{3}:0.009$
Piezoresistive [42]	GO/Gr	-	$S_{1}\sim S_{3}:0.032$
Piezoresistive [43]	JGF	-	$S_{1}\sim S_{3}:0.005$
Capacitive [44]	PDMS	-	$S_{1}\sim S_{2}:0.42$, $S_{3}:0.04$
Capacitive [45]	PDMS	-	$S_{1}\sim S_{2}:0.55$, $S_{3}:0.15$
Capacitive [46]	PDMS/Graphene	-	$S_{1}:\;0.33,$ $S_{2}\sim S_{3}:$0.007

**Table 4:** Comparison of response current ratio according to TFT back gate voltage control.

Gate Voltage [V]	$\mathrm{Ratio}\;\mathrm{of}\;\mathrm{Response}\;\mathrm{Current},\;∆I_{0}/I\;[\%]$		
	Thin Film	Film Annealing	NRs Stacked
0	8.45	13.9	50.6
1	6.14	10.0	45.4
2	6.09	6.04	40.7
3	5.64	3.52	33.7
4	3.63	2.11	27.4
5	2.67	3.28	21.8

**Table 5:** TFT parameter extraction.

	Thin Film		NRs Stacked	
	No Press	Press	No Press	Press
$V_{th}$ [V]	−13.9	−13.6	−4.5	−3.62
SS [V/dec]	6.23	6.92	7.29	6.80
$I_{on/off}$	1.44 × 10^6^	1.16 × 10^6^	8.37 × 10^5^	1.77 × 10^6^
Mobility $[{\mathrm{cm}}^{2}/V\;s$]	5.71	3.83	2.99	0.98

## Data Availability

Not applicable.
